# Mitoguardin-1 and -2 promote maturation and the developmental potential of mouse oocytes by maintaining mitochondrial dynamics and functions

**DOI:** 10.18632/oncotarget.6713

**Published:** 2015-12-21

**Authors:** Xiao-Man Liu, Yong-Ping Zhang, Shu-Yan Ji, Bo-Tai Li, Xuejun Tian, Dali Li, Chao Tong, Heng-Yu Fan

**Affiliations:** ^1^ Life Sciences Institute and Innovation Center for Cell Signaling Network, Zhejiang University, Hangzhou, China; ^2^ Shanghai Key Laboratory of Regulatory Biology, Institute of Biomedical Sciences and School of Life Sciences, East China Normal University, Shanghai, China

**Keywords:** mitochondrion, ROS, mtDNA copy number, oocyte meiosis, female infertility, Pathology Section

## Abstract

Mitochondrial dynamics change mitochondrial morphological features and numbers as a part of adaptive cellular metabolism, which is vital for most eukaryotic cells and organisms. A disease or even death of an animal can occur if these dynamics are disrupted. Using large-scale genetic screening in fruit flies, we previously found the gene mitoguardin (*Miga*), which encodes a mitochondrial outer-membrane protein and promotes mitochondrial fusion. Knockout mouse strains were generated for the mammalian *Miga* homologs *Miga1* and *Miga2*. *Miga1/2^−/−^* females show greatly reduced quality of oocytes and early embryos and are subfertile. Mitochondria became clustered in the cytoplasm of oocytes from the germinal-vesicle stage to meiosis II; production of reactive oxygen species increased in mitochondria and caused damage to mitochondrial ultrastructures. Additionally, reduced ATP production, a decreased mitochondrial-DNA copy number, and lower mitochondrial membrane potential were detected in *Miga1/2^−/−^* oocytes during meiotic maturation. These changes resulted in low rates of polar-body extrusion during oocyte maturation, reduced developmental potential of the resulting early embryos, and consequently female subfertility. We provide direct evidence that MIGA1/2-regulated mitochondrial dynamics is crucial for mitochondrial functions, ensure oocyte maturation, and maintain the developmental potential.

## INTRODUCTION

In mammalian females, oocytes are arrested at the germinal vesicle (GV) stage of meiosis I and are stored in ovarian follicles for years or even decades. These GV stage-arrested oocytes have a low metabolic rate in order to maintain the stability of their inheritance materials (including genomic DNA and mitochondrial DNA [mtDNA] as well as other cellular organelles) and to accurately transmit them to the offspring [1]. During meiotic maturation, however, oocyte energy metabolism increases due to the requirements of multiple physiological events, such as GV breakdown (GVBD), spindle formation, chromosome alignment and separation, and polar-body extrusion (PBE) [2]. In addition, early embryonic development and implantation are also energy-consuming processes. Thus, numerous mitochondria develop and are stored in an oocyte's cytoplasm. They provide energy by producing ATP and by assisting with spindle assembly and orientation during meiotic maturation and early embryo cleavages [3, 4].

Normal mitochondrial function is crucial for successful oocyte maturation and early embryonic development [5-7]. Mitochondrial dysfunction has been implicated in increased formation of abnormal spindles and chromosome aneuploidy in oocytes of mice fed a high-fat diet; these changes may account for the infertility observed in obese women [8, 9]. Maternal diabetes results in defective oocyte meiosis by disrupting mitochondrial structures and metabolic functions [10]. Insulin resistance was found to disrupt mitochondrial function by reducing mtDNA copy numbers and ATP levels in mouse MII oocytes. This mechanism may contribute to the low fertility rate in diabetic women [11]. Mitochondria aggregate in aged-oocyte cytoplasm and synthesize a reduced amount of ATP, and these defects can prevent oocyte maturation and ovulation and ultimately may result in female reproductive failure [5, 12, 13]. To date, however, there have been few studies on the regulation of mitochondrial dynamics during oocyte meiosis and embryonic development.

We recently identified a gene that encodes for a mitochondrial protein in *Drosophila* designated *mitoguardin* (*Miga*; Zhang and Liu et al., Molecular Cell, 2016 Jan, in press). It has two poorly studied homologs (family with sequence similarity 73, members A and B, *Fam73a* and *Fam73b*) in vertebrates, which we renamed *Miga1* and *Miga2*. MIGA1 and MIGA2 are nucleus-encoded proteins that are localized to the outer membrane of mitochondria. MIGA1/2 promote mitochondrial fusion by interacting with the mitochondrial outer-membrane protein Mito-PLD [14]. The latter is a signaling molecule involved in cardiolipin hydrolysis (in the synthesis of phosphatidic acid) and promotes mitochondrial fusion [14].

To identify the *in vivo* function of the *Miga1/2*, we generated *Miga1* and *Miga2* single- and double-knockout (KO) mouse strains and found that these KO females are subfertile. MIGA1/2 regulate mitochondrial dynamics and functions during oocyte meiosis and embryonic development. These results provide new insights into the mechanisms of mitochondrial fusion and may help to identify new therapeutic targets in female sterility of unknown etiology.

## RESULTS

### Inhibition of mitochondrial function blocked oocyte maturation

**Figure 1:**
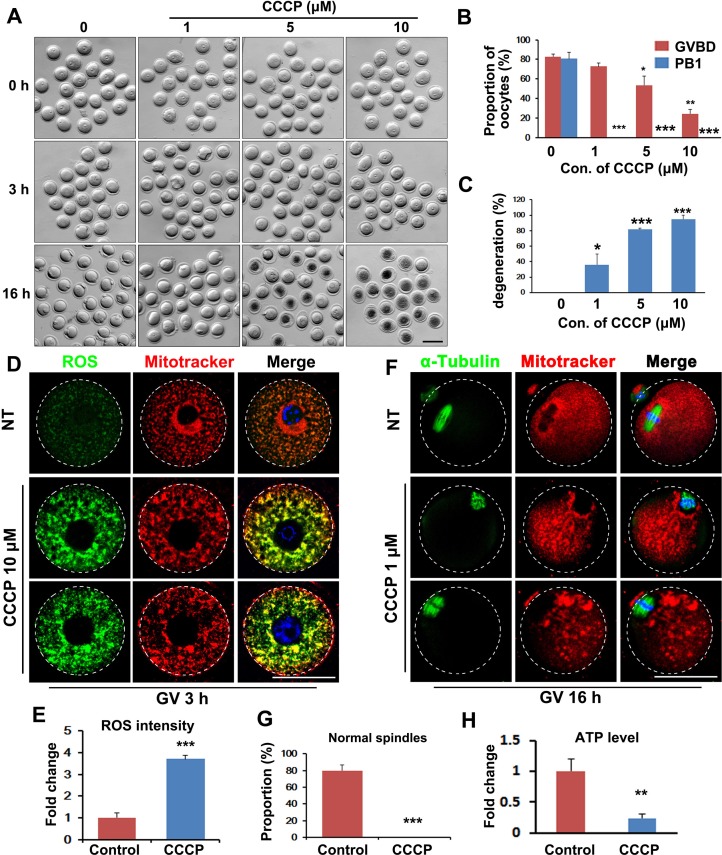
Inhibition of mitochondrial function by carbonylcyanide-m-chlorophenylhydrazone (CCCP) blocks oocyte maturation **A.** Representative images of CCCP-treated oocytes. Scale bar = 100 μm. **B.** Effects of CCCP on germinal-vesicle breakdown (GVBD) and polar-body extrusion (PBE) rates of cultured oocytes. **C.** Effects of CCCP on degeneration rates of cultured oocytes. **D.** Fluorescent staining showing increased reactive oxygen species (ROS) levels (green) and clustered mitochondria (red) in CCCP-treated oocytes. Scale bar = 50 μm. **E.** Quantification of ROS signals in oocytes. **F.** Fluorescent staining showing disrupted spindles (green) and clustered mitochondria (red) in CCCP-treated oocytes. Scale bar = 50 μm. **G.** The proportion of normal spindles in control and CCCP-treated oocytes, after 16 h of culture. H. ATP contents in control and CCCP-treated oocytes.

To assess mitochondrial function during oocyte meiosis, we incubated oocytes with the mitochondria-targeted compound carbonylcyanide-m-chlorophenylhydrazone (CCCP), which is a proton gradient uncoupler, i.e., it disrupts mitochondrial ATP production. After CCCP treatment, oocytes meiotic maturation was arrested. More than 80% of the control oocytes resumed meiosis within 3 h of *in vitro* culture, and this process was characterized by GVBD, whereas oocytes treated with CCCP showed reduced GVBD rates, in a dose-dependent manner (in the range 1-10 μM CCCP; Figure 1A and 1B). In addition, CCCP-treated oocytes failed to release polar body 1 (PB1; Figure 1A and 1B) and degenerated in a dose-dependent manner (Figure 1A and 1C).

Staining with fluorescent probes showed that mitochondria in the oocytes became clustered after CCCP treatment (10 μM, 3 h), and reactive oxygen species (ROS) levels increased (1 μM, 16 h. Figure 1D and 1E). Meiotic spindle formation was also compromised by CCCP treatment (Figure 1F-1G). CCCP reduced ATP content of oocytes after incubation for 3 h (Figure 1H). Collectively, these results indicated that oocyte meiosis requires active ATP production by mitochondria.

### *Miga1* and *Miga2* knockout females showed decreased quality of oocytes

**Figure 2:**
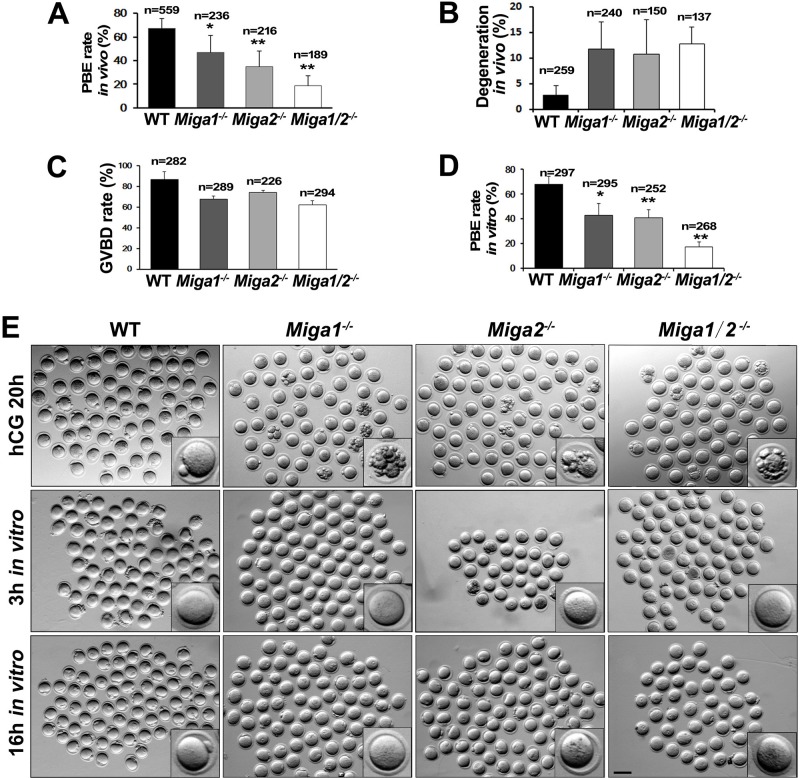
*Miga1/2^−/−^* mice show reduced fertility and defects in oocyte development **A.** Polar body 1 (PB1) extrusion (PBE) rates of the oocytes ovulated in mice with the indicated genotypes. Total numbers (n) of observed oocytes are indicated. **B.** Degeneration rates of oocytes ovulated in mice with the indicated genotypes. Total numbers (n) of oocytes observed are indicated. **C.**-**D.** GVBD and PBE rates of cultured oocytes isolated from the mice of the indicated genotypes. Total numbers (n)of cultured oocytes are indicated. **E.** Representative images of ovulated (the upper row) and cultured oocytes (the middle and lower rows) of the indicated genotypes. Scale bar = 100 μm.

The ovulated oocytes from *Miga1^−/−^*, *Miga2^−/−^*, and *Miga1/2^−/−^* mice had significantly lower-than-normal PBE rates (Figure 2A and 2E). Approximately 10% of the oocytes that were ovulated by the KO mice degenerated already at 16 h after human chorionic gonadotropin (hCG) injection (Figure 2B and 2E).

To confirm the low quality of the oocytes from the KO mice and to rule out the effects of other factors *in vivo*, fully grown GV stage oocytes were collected from antral follicles and cultured *in vitro*. Oocytes from the KO mice showed slightly decreased GVBD rates, after 3 h of culture (Figure 2C and 2E). In line with the *in vivo* results, PB1 extrusion rates were significantly lower in KO oocytes than in WT oocytes after 16 h of culture (Figure 2D and 2E). The oocytes from *Miga1/2* KO mice had a phenotype (low PBE rate and high degeneration rate) similar to that of the oocytes treated with CCCP *in vitro*, suggesting that the *Miga1/2* KO mice have low quality of oocytes because of mitochondrial defects.

### Mitochondrial functions were impaired in *Miga1/2*-deleted oocytes

**Figure 3:**
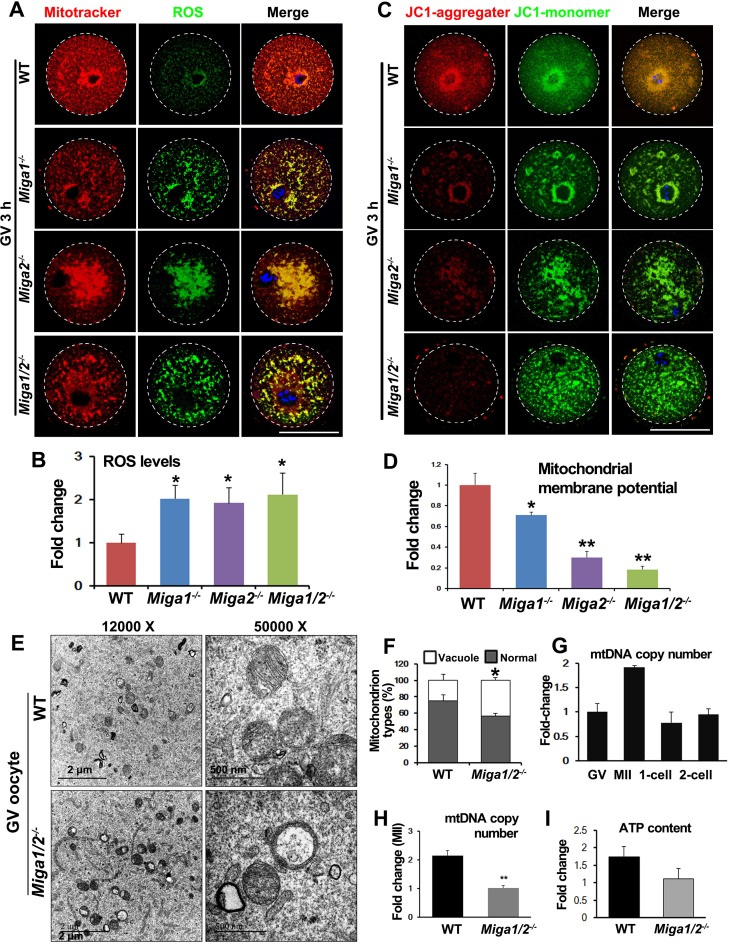
Mitochondria in oocytes of *Miga1/2* knockout mice show an altered distribution and impaired functions **A.** Mitochondria distributions and reactive oxygen species (ROS) levels in oocytes isolated from mice of the indicated genotypes, and fixed at 3 h after GVBD. Scale bar = 50 μm. **B.** Quantification of ROS levels in oocytes of the indicated genotypes. **C.** The mitochondrial membrane potential (MMP) in oocytes isolated from mice of the indicated genotypes, according to JC-1 staining. Scale bar = 50 μm. **D.** Quantitative results on the relative MMP levels in oocytes of the indicated genotypes. **E.** Electron microscopy results showing that mitochondria had lost cristae and formed vacuoles in *Miga1/2^−/−^* oocytes. **F.** Quantitative results on the numbers of mitochondria with vacuoles in wild-type (WT) and *Miga1/2^−/−^* oocytes. **G.** Relative mtDNA copy numbers in WT oocytes at indicated stages. **H.**-**I.** Relative mtDNA copy numbers (H) and ATP levels (I) in WT and *Miga1/2^−/−^* oocytes at the MII stage.

Because *Miga1* and *Miga2* are important for mitochondrial functions in somatic cells, we assessed mitochondrial functions in the oocytes of *Miga1/2^−/−^* mice. Mitotracker staining showed that mitochondria in WT oocytes were evenly distributed throughout the oocyte cytoplasm but partially gathered around spindles (Figure 3A). In contrast, in the cytoplasm of *Miga1^−/−^*, *Miga2^−/−^*, and *Miga1/2^−/−^* oocytes, mitochondria were aggregated. These morphological changes resulted in increased ROS levels (Figure 3A and 3B). ROS signals were colocalized with mitochondria, suggesting that ROS that were generated in the mitochondria accumulated and were caged in the mitochondria, thereby possibly adversely affected mitochondrial functions or ultrastructures.

Then, we tested the mitochondrial membrane potential (MMP, _Δ_ψ) using 5,5,6,6-tetrachloro-1,1,3,3-tetraethyl-β-benzimidazolylcarbocyanine iodide (JC-1) staining. JC-1 is a compound that selectively enters the mitochondria and reversibly changes from monomers (green fluorescence) to aggregates (orange fluorescence) when the MMP is high on the inner mitochondrial membrane. The ratio of orange to green fluorescence reflects the MMP level in cells. In this assay, the MMP of *Miga1/2^−/−^* oocytes was significantly reduced as compared to that of WT oocytes, indicating that in the *Miga1/2^−/−^* oocytes, mitochondrial activity decreased (Figure 3C and 3D).

High levels of ROS can cause damage to the mitochondria [15, 16], and the decreased MMP usually correlates with alteration of the mitochondrial cristae structure [17]. Electron-microscopic images showed that in WT oocytes, mitochondria were generally evenly distributed with normal aligned cristae. In *Miga1/2^−/−^* oocytes, however, mitochondria were tethered closely to each other (Figure 3E). More mitochondria lost their cristae and contained large vacuoles than did mitochondria in WT oocytes (43.3% in *Miga1/2^−/−^* oocytes *versus* 25% in WT oocytes, Figure 3F).

The mtDNA copy number is also an indicator of mitochondrial activity. We quantified mtDNA copy numbers in WT oocytes and embryos at the GV, MII, 1-cell, and 2-cell stages and found that mtDNA copy numbers increased nearly 2-fold from the GV to MII stage (Figure 3G). The mtDNA copy numbers in MII-arrested *Miga1/2^−/−^* oocytes were approximately half of those in WT oocytes (Figure 3H). In addition, ATP production in *Miga1/2^−/−^* oocytes was correspondingly reduced (Figure 3I).

### Precision of chromosome separation was disrupted in *Miga1/2*-deleted oocytes

**Figure 4:**
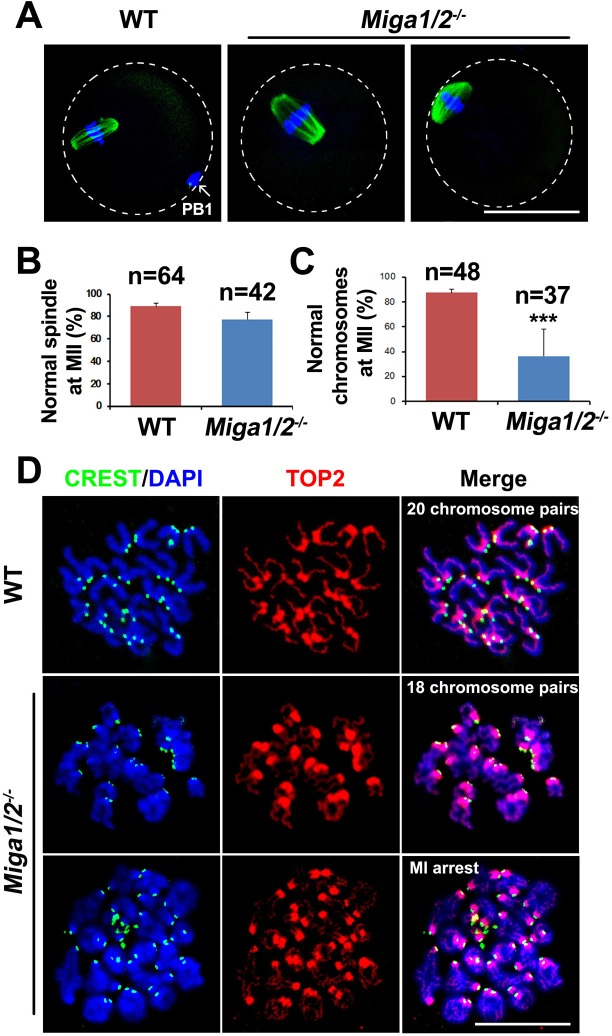
Spindle assembly and chromosome separation in wild-type (WT) and *Miga1/2^−/−^* oocytes **A.** Confocal microscopy images showing spindles in WT and *Miga1/2^−/−^* oocytes after 16 h of culture. The arrow indicates polar body 1 (PB1). Scale bar = 50 μm. **B.** and **C.** The percentage of oocytes with normal spindles (B) and normal chromosome numbers (C) after 16 h of culture. Total numbers (n) of the examined oocytes are indicated. **D.** Representative chromosome spreads from WT and *Miga1/2^−/−^* oocytes after 16 h of culture. Chromosome configurations are shown by immunofluorescent staining for TOP2 (red) and CREST (green). DNA was labeled by 4′,6-diamidino-2-phenylindole (DAPI, blue). Scale bar = 50 μm.

*Miga1/2^−/−^* oocytes showed decreased PBE rates (Figure 2C and 2F-2G) during meiotic maturation. Unexpectedly, most *Miga1/2^−/−^* oocytes could form spindles although they appeared thicker than those of WT oocytes (Figure 4A and 4B). However, 63.6% of oocytes ovulated by *Miga1/2^−/−^* mice had abnormalities in the chromosome number or configuration, whereas only ~12.5% of chromosomes were abnormal in WT oocytes (Figure 4C and 4D). Normal chromosomes in MII stage oocytes can be defined as 20 pairs of sister chromatids that were attached at centromeres; in contrast, in *Miga1/2^−/−^* oocytes, chromosome numbers ranged from 14 to 18 pairs. Some *Miga1/2^−/−^* oocytes were arrested at the MI stage with 20 pairs of homologous chromosomes (i.e., 80 chromatids; Figure 4C).

### *Miga1/2*-deleted oocytes have a poor development potential after fertilization

**Figure 5:**
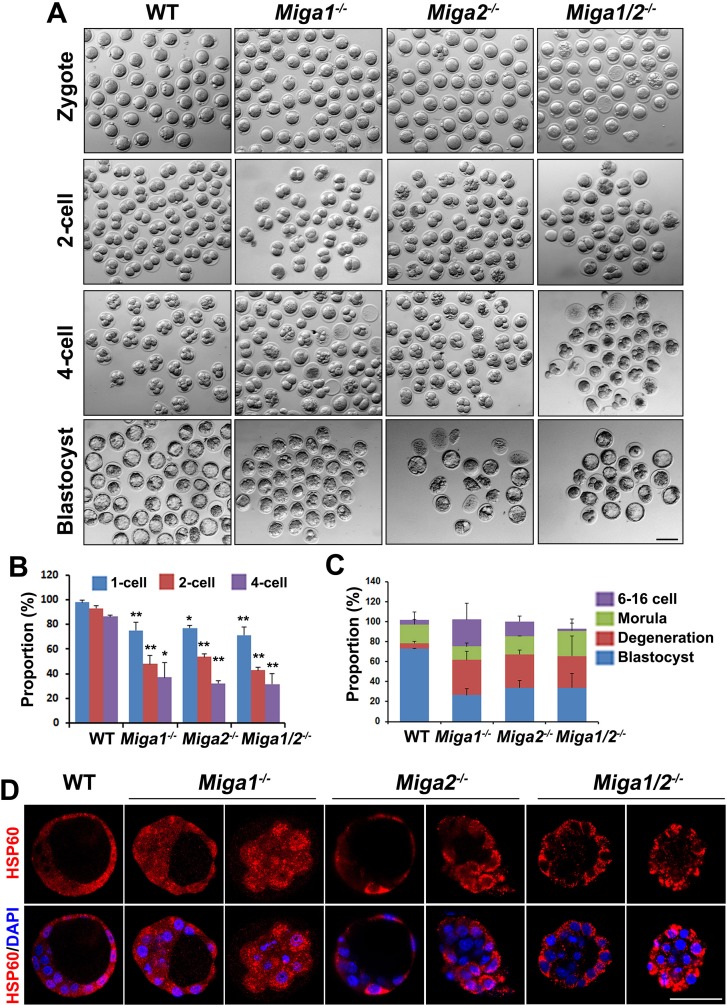
Early embryos derived from *Miga1/2^−/−^* oocytes have a low developmental potential **A.** Early embryos derived from oocytes of the indicated genotypes. Scale bar = 100 μm. **B.** Proportions of the indicated embryos from female mice of the indicated genotypes at day 0.5, 1.5, 2.0 after mating with wild-type (WT) males. **C.** Proportions of 6- to 16-cell embryos, morulae, blastocysts, and degenerated embryos from female mice of the indicated genotypes on day 4 after mating with WT males. **D.** The distribution of mitochondria in embryos from female mice of the indicated genotypes on day 4 after mating with WT males, as indicated by immunofluorescent staining for HSP60. Scale bar = 50 μm.

The zygotes derived from *Miga1^−/−^*, *Miga2^−/−^*, and *Miga1/2^−/−^* oocytes all had a poor developmental potential as compared to embryos from WT females (Figure 5A-5C). Although 72.8% of zygotes of WT females developed to blastocysts on day 4 after coitus, this rate was only 30.4% in *Miga1/2^−/−^* females (Figure 5C). Most of the abnormal embryos degenerated, particularly during the development from the 1-cell to 4-cell stage (Figure 5A). Mitochondria were clustered in the cytoplasm of the embryos from *Miga1^−/−^*, *Miga2^−/−^*, and *Miga1/2^−/−^* mice, especially in the embryos arrested at the morula stage, according to immunostaining for the mitochondrial protein HSP60 (Figure 5D). These results indicated that the abnormal mitochondrial distribution in blastomeres that is caused by maternal deletion of MIGA1/2 may contribute to the failure of subsequent embryonic development.

### ATP and vitamin C (Vc) partially reversed the defects of *Miga1/2*-deleted oocytes

**Figure 6:**
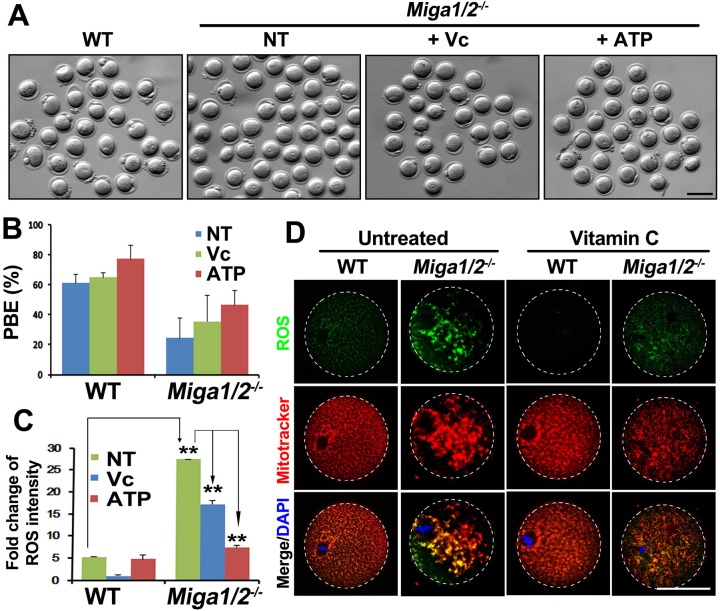
Vitamin C partially reverses mitochondrial defects in *Miga1/2^−/−^* oocytes **A.**-**B.** Images (A) and polar-body extrusion (PBE) rates (B) of oocytes after 12 h of culture, with or without addition of vitamin C or ATP to the media. Scale bar = 100 μm. **C.** Relative reactive oxygen species (ROS) levels of oocytes after 12 h of culture, with or without addition of vitamin C or ATP to the media. **D.** Confocal microscopy results showing ROS signals and mitochondrial distributions in oocytes after 12 h of culture, with or without addition of vitamin C or ATP to the media. Scale bar = 50 μm.

Vc is an antioxidant that can reduce ROS levels in plant cells [18-20] and mouse embryonic fibroblasts [21]. Because deletion of MIGA1/2 in oocytes caused mitochondrial damage and disrupted mitochondrial dynamics, thus resulting in lower ATP levels and ROS accumulation in oocytes, we tested whether these defects could be reversed by Vc, at least partially. Vc treatment (25 μg/mL) increased PBE rates in both WT oocytes and *Miga1/2^−/−^* oocytes, but more significantly in the latter (Figure 6A and 6B). In addition, Vc treatment remarkably reduced ROS levels and partially rescued the normal mitochondrial distribution in *Miga1/2^−/−^* oocytes (Figure 6C and 6D). Meanwhile, ATP supplementation also partially reversed the PBE defects and reduced ROS levels in *Miga1/2^−/−^* oocytes (Figure 6A-6C).

### Oocyte meiosis was arrested in conditional knockout mice

**Figure 7:**
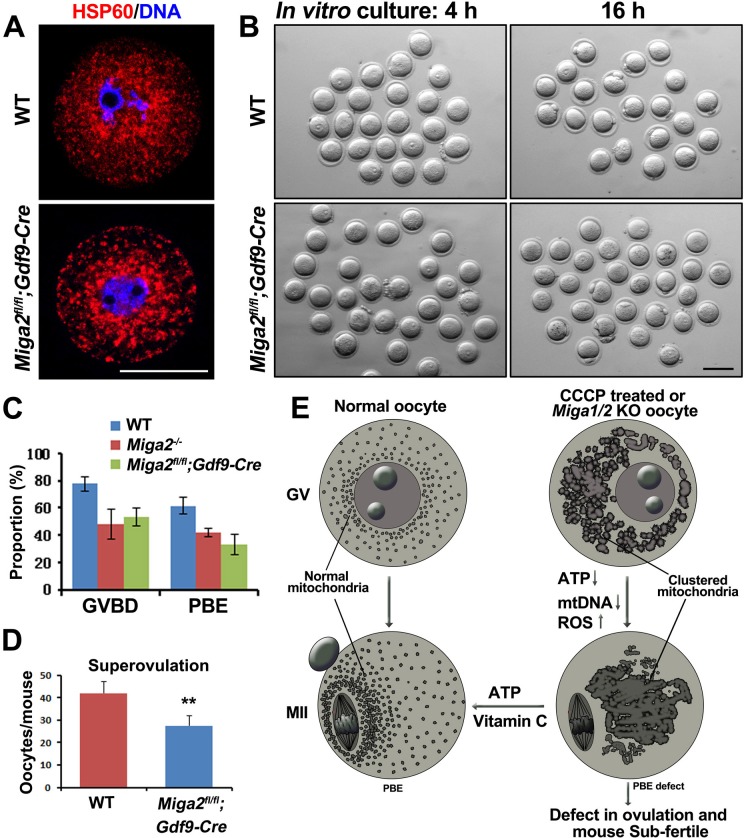
Phenotype analyses of mice with an oocyte-specific *Miga2* knockout **A.** Mitochondrial distributions in germinal vesicle (GV) stage oocytes of wild-type (WT) and *Miga2^flox/flox^Gdf9-Cre* mice (oocyte-specific *Miga2* KO), as indicated by immunofluorescent staining for HSP60. Scale bar = 50 μm. **B.** Oocytes of WT and *Miga2^flox/flox^Gdf9-Cre* mice after 4 and 16 h of culture. Scale bar = 100 μm. **C.** *In vitro* germinal-vesicle breakdown (GVBD) and polar-body extrusion (PBE) rates of oocytes from mice of the indicated genotypes. **D.** Average numbers of oocytes being ovulated by mice of the indicated genotypes. **E.** Summary: Deletion of *Miga1*/*2* or CCCP treatment results in mitochondrial clustering, reduces ATP levels and mitochondrial DNA (mtDNA) copy numbers, and increases reactive oxygen species (ROS) levels in oocytes; these changes cause meiotic and developmental defects. Addition of vitamin C or ATP to the oocyte culture medium partially reverses these problems.

To determine whether the fertility in *Miga1/2^−/−^* mice was reduced by oocyte-related factors, we generated oocyte-specific *Miga2* KO mice (*Miga2^flox/flox^Gdf9-Cre* mice) and analyzed the oocyte meiosis *in vitro*. Oocytes from the *Miga2^flox/flox^Gdf9-Cre* mice also contained aggregated mitochondria (Figure 7A) and had defects in GVBD and PBE that were similar to those in the *Miga2^−/−^* oocytes (Figure 7B and 7C). In addition, superovulation in *Miga2^flox/flox^Gdf9-Cre* mice still produced a reduced number of ovulated oocytes (Figure 7D), suggesting that it was the oocyte degeneration that contributed to the lower number of superovulated oocytes in *Miga2^flox/flox^Gdf9-Cre* mice.

## DISCUSSION

Mitochondria undergo frequent morphological changes because of fission and fusion. Malfunctioning of the mitochondrial fusion and fission causes various human developmental disorders, including reduced fertility. Mitochondrial functions strongly correlate with mitochondrial structure and morphology [22, 23]. When metabolism requires that mitochondria produce large amounts of ATP, mitochondria form wide long cristae, and mitochondria become elongated and connected in a persistent rapidly dynamic manner. In contrast, when mitochondrial functions are disrupted or slowed down (lowered metabolic rate), mitochondria form narrow short cristae and become fragmented or clustered depending on the cell type [24, 25].

In a recent study (Zhang *et al*., manuscript submitted), we uncovered an evolutionarily conserved new family of outer-membrane mitochondrial proteins: MIGA, which promotes mitochondrial fusion in both *Drosophila* and mice (Zhang *et al*., manuscript submitted). MIGA interacts with MitoPLD, thus stabilizing MitoPLD and promoting its self-association affinity and driving mitochondrial fusion. Loss of *Miga* in flies caused neurodegeneration. MIGA proteins are also linked to the fat metabolism in mammals (Zhang *et al*., submitted to Molecular Cell, under revision). In the present study, we further demonstrate that MIGA1 and MIGA2 are involved in oocyte meiosis, maturation, and developmental potency.

The mitochondria-targeted compound CCCP uncouples the MMP, disrupts ATP production, and results in mitochondrial fragmentation; these effects are similar to the phenotypes observed after deletion of *Miga1/2* in mouse embryonic fibroblasts [16, 26]. In the present study, CCCP arrested oocyte meiosis at the MI stage by inducing oocyte degeneration after oocyte GVBD. CCCP treatment strongly reduced ATP synthesis; in addition, elevated amounts of ROS were detected in clustered mitochondria, and this change contributed to abnormal spindle formation in the oocytes incubated with CCCP. The phenotype of *Miga1/2^−/−^* oocytes was similar to that of CCCP-treated oocytes *in vitro*, indicating that MIGA1/2 may regulate mitochondrial activities by controlling mitochondrial morphological remodeling.

The *Miga1/2^−/−^* oocytes showed defective mitochondrial dynamics and clustered mitochondria in the cytoplasm; these changes were probably linked to the high ROS levels inside these mitochondria. This high concentration of ROS may have reduced the mitochondrial membrane potential, reduced mtDNA copy numbers, and disrupted mitochondrial metabolism (ATP production). Furthermore, the lack of ATP may have contributed to the severe damage to the ultrastructures of mitochondrial cristae because most mitochondria lost their cristae and contained large vacuoles.

PBE requires energy for separation of chromosomes and for division of the cytoplasm into 2 parts. The lack of ATP and an environment with a high ROS level may contribute to the failure of oocyte PBE. In addition, strong oxidative stress, such as that caused by H_2_O_2_, has been implicated in disruption of spindle formation, particularly during the MII stage of oocytes, by reducing the amount of mitochondria-derived ATP [27]. Once spindle formation or movement is disrupted, this change is very likely to cause abnormal chromosome separation and to increase the chances of aneuploidy. Additionally, ROS stress can reduce mtDNA copy numbers, thereby decreasing the ATP level and producing mutations in mtDNA [28, 29].

Vc is an efficient antioxidant and can reduce high ROS concentrations [20, 21]. We demonstrated that Vc treatment of oocytes reduced their ROS levels and partially reversed the defects in oocyte meiosis. Although Vc rescued mitochondrial morphology, it only partially reversed the defect in oocyte PBE in *Miga1/2^−/−^* mouse oocytes (Figure 7E). These results suggest that the disordered mitochondrial dynamics that resulted in the defective PBE cannot be completely reversed simply by reducing ROS levels.

In summary, we demonstrated that the nucleus-encoded mitochondrial proteins MIGA1 and MIGA2 are required for mitochondrial dynamics and functions in oocytes, and promote their developmental potential. Therefore, this study not only provides evidence of the physiological importance of mammalian MIGA proteins but also provides new insights into female infertility.

## MATERIALS AND METHODS

### Mice

*Miga1* KO mice were produced by TALEN from the FVB/N strain as described previously [30]. *Miga2* KO-first mice were purchased from the Jackson Laboratory. *Miga2^flox/flox^* mice were generated by crossing *Miga2* KO-first mice with *FLPase* mice. *Miga2^flox/flox^Gdf9-Cre* female mice were created by crossing *Miga2^flox/flox^Gdf9-Cre* male mice with *Miga2^flox/flox^* females. Mice were raised in an environment with stable temperature (20-22°C), 12/12 h light/dark cycle, 50-70% humidity, and food and water provided manually regularly. Animal care and experimental procedures were in accordance with the Animal Research Committee guidelines of Zhejiang University. The *Miga2^flox/flox^Gdf9-Cre* mice have a C57BL/6J background. And all the mice in the experiments had a mixed background of FVB/N and C57BL/6J strains.

### Oocyte culture

Twenty-one-day-old females were injected with 5 IU pregnant mare serum gonadotropin (Ningbo Sansheng Pharmaceutical Co., Ltd., China), and after 44 h, the mice were euthanized and the ovaries were chopped in a culture dish. Oocytes at the GV stage were cultured in drops of the M16 medium (M7292; Sigma-Aldrich) covered with mineral oil (M5310; Sigma-Aldrich) at 37°C in a humidified atmosphere containing 5% of CO_2_.

### ROS detection

ROS were detected by means of the ROS detection assay kit (Beyotime) according to the manufacturer's instructions. In short, oocytes were stained in 2′,7′-dichlorofluorescin diacetate (DCFH-DA) in the M2 medium for 20 min at room temperature, washed, mounted on a glass slide, and examined under a confocal laser scanning microscope (Zeiss LSM 710, Carl Zeiss AG, Germany).

### An immunofluorescence assay of the mouse oocytes

Oocytes were fixed in 4% paraformaldehyde (PFA) in PBS and incubated with 0.2% Triton X-100 in PBS for 30 min. After blocking the cells with 1% BSA in washing buffer (PBST: PBS with 0.1 % Triton X-100), we incubated oocytes with primary antibodies buffered in the blocking solution. After three washes, oocytes were incubated with secondary antibodies and counterstained with 4′,6-diamidino-2-phenylindole (DAPI). The oocytes were mounted on glass slides using SlowFade^®^ Gold Anti-fade Reagent (Life Technologies) and examined under a confocal microscope.

### Superovulation and fertilization

For superovulation, female mice (21-23 days) were injected intraperitoneally with 5 IU pregnant mare serum gonadotropin and 44 h later with 5 IU hCG (Ningbo Sansheng Pharmaceutical Co., Ltd., China). After 16 h, cumulus cell-oocyte complexes were excised from the ampullar region of oviducts and the numbers of oocytes were counted. The oocytes were examined and photographed by means of a Nikon SMZ1500 stereoscope.

To obtain early embryos, female mice were mated with 10- to 12-week-old WT males overnight. Successful mating was confirmed by the presence of vaginal plugs. Zygotes and 2-cell and 4-cell embryos were harvested from oviducts at indicated time points after the hCG injection. The blastocysts were collected from the uterus in the evening of day 4 after mating.

### Quantification of mtDNA copy number

A single oocyte was lysed in 5 μL of lysis buffer (50 mM Tris-HCl pH 8.0, 1 mM EDTA, 0.5% Tween-20, 100 mg/mL protease K) in a PCR tube and incubated in 55°C for 2 h, then at 95°C for 10 min, after which the sample was directly used in RT-PCR with the mtDNA-specific primers (Forward: 5′-TACCTCACCATCTCTTGCTA-3′; Reverse: 5′-CCACATAGACGAGTTGATTC-3′). The data on the mtDNA were normalized to β-globin.

### Luminescence testing for ATP quantification

ATP content of oocytes was determined with the ATP Testing Assay Kit (Beyotime) according to the manufacturer's instructions. Briefly, 50 oocytes were lysed in ATP lysis buffer (from the kit) and centrifuged at 12,000 ×*g* for 10 min. Supernatants were mixed with testing buffer, and ATP concentrations were measured on a luminescence detector.

### Transmission electron microscopy

Ovaries were fixed in 2.5 % glutaraldehyde and 1% osmic acid. After that, the ovaries were dehydrated and incubated in the embedding medium overnight. The next day, they were embedded in the embedding medium and heated at 65°C for 2-3 days. The samples were cut into sections of 70-90 nm on an ultramicrotome (Leica). The sections were stained by a lead citrate solution and analyzed under an electron transmission microscope (HT7700, Hitachi).

### Preparation of chromosome spreads

Oocytes were harvested at the MII stage, and the zona pellucida was removed by the acidic M2 medium (pH 2.0). Then, the oocytes were fixed in the CS solution (1% PFA, 0.15% Triton X-100, and 3 mM dithiothreitol) on glass slides. Slides were air dried and immunofluorescence was performed as described above for oocytes.

### Western blotting

Protein samples were harvested in 1×SDS loading buffer and separated by SDS-PAGE and electrophoretically transferred to polyvinylidene fluoride membranes (Millipore). After incubation with primary antibodies and a horseradish peroxidase-linked secondary antibody, bands on the membranes were detected by the Enhanced Chemiluminescence Detection Kit (Amersham) and an X-ray film.

### Statistical analysis

The results are presented as mean ± SD; each experiment included at least three replicates and was repeated at least three times. Group comparisons were conducted by two-tailed unpaired Student's *t* tests. Differences with *P* values <0.05 were considered significant.
